# Selection for social genetic effects in purebreds increases growth in crossbreds

**DOI:** 10.1186/s12711-021-00609-2

**Published:** 2021-02-12

**Authors:** Birgitte Ask, Lizette Vestergaard Pedersen, Ole Fredslund Christensen, Hanne Marie Nielsen, Simon P. Turner, Bjarne Nielsen

**Affiliations:** 1 Danish Pig Research Centre, Danish Agriculture & Food Council F.M.B.A, SEGES, Axeltorv 3, 1609 AxelborgCopenhagen V, Denmark; 2 Center for Quantitative Genetics and Genomics, Aarhus University, Blichers Allé 20, 8830 Tjele, Denmark; 3 Animal & Veterinary Sciences, SRUC, Roslin Institute Building, Easter Bush, Midlothian, EH25 9RG UK

## Abstract

**Background:**

Average daily gain (ADG) in pigs is affected by both direct and social genetic effects (SGE). However, selection for SGE in purebreds has not conclusively been shown to improve ADG in crossbreds, and it is unknown whether SGE in purebreds are equal to those in crossbreds. Moreover, SGE may reflect dominance related behaviour, which is affected by the variation in body weight within a group. Therefore, we hypothesized that (a) there is a positive effect of parent average SGE estimated in purebred pigs on phenotypic ADG in crossbred offspring, and (b) there is an interaction between SGE on ADG and standard deviation in starting weight of pigs within the group. We also hypothesized that (c) social genetic variance for ADG exists in crossbred pigs, and (d) there is a favourable genetic correlation between SGE on ADG in purebred and crossbred pigs.

**Results:**

We found a statistically significant interaction between the standard deviation in starting weight and SGE within groups, and conditioning on the mean standard deviation in starting weight, we found a favourable regression coefficient (0.37 ± 0.21) of ADG in crossbreds on SGE in purebreds. Variances for SGE were small in both Landrace (L) and Yorkshire (Y), and higher for SGE in both the dam and sire component of crossbred YL. The genetic correlations between SGE in purebreds and the dam or sire component of SGE in crossbreds were also favourable (0.52 ± 0.48 and 0.34 ± 0.42, respectively), although not significantly different from 0.

**Conclusions:**

We confirmed that there is a positive effect of SGE estimated using purebred information on phenotypic ADG in crossbreds, and that the largest effect is achieved when the within-group variation in starting weight is small. Our results indicate that social genetic variance in crossbreds exists and that there is a favourable genetic correlation between social genetic effects in purebreds and crossbreds. Collectively, our results indicate that selection for SGE on ADG in purebreds in a nucleus farm environment with little competition for resources can improve ADG in crossbreds in a commercial environment.

## Background

Average daily gain (ADG) is an economically important trait in commercial pig production, and therefore also in many pig breeding programs. The direct heritable effect on ADG, which is caused by the genetic effect of the focal individual, is complemented by additional heritable effects on ADG caused by the genetic effects of other group members acting on the phenotype of the focal individual. Due to these so-called social genetic effects (SGE) [1, 2], the total heritable variation is higher than the direct heritable variation [3, 4]. Hence, the response to selection for a combination of direct genetic effects (DGE) and SGE for ADG is expected to be higher than if selection is based on DGE alone. However, in practice, this expectation has not yet been conclusively confirmed. The predictive ability of combined DGE and SGE reflects the expected accuracy of selection and ought to be higher than the predictive ability of DGE alone. Yet, reported predictive abilities of combined DGE and SGE have not consistently confirmed this expectation when using different models [4] and populations [5]. Likewise, previous single-generation selection experiments have been inconclusive. Hong et al. [6] showed that groups of pigs selected for high SGE had higher ADG than groups of pigs selected for low SGE. Conversely, Camerlink et al. [7] did not detect such a difference. Confirmation that selection on SGE results in improved selection response is important before including SGE in selection indices, and therefore, it is still essential to verify that selection based on SGE for ADG will improve phenotypic ADG.

At least four reasons may explain these inconclusive selection experiment results. First, the sample size and statistical power to estimate genetic effects accurately could be insufficient, which is in fact the conclusion of Camerlink et al. [7]. In addition, Hong et al. [6] had an even smaller sample size (70 individual pigs and 14 groups in total). Second, genotype-by-environment interactions may cause reranking of the effects of genotypes between experimental and selection environments [8]; hence, selection for SGE in one environment may not yield the expected selection response in another environment. Selection and recording were performed in the same environment in the experiment of Hong et al. [6], whereas in the experiment of Camerlink et al. [7], recording was performed in an experimental environment, which differed from the selection environment. In pig breeding, the selection environments (nucleus farms) typically differ from commercial production environments with regards to group size, space allowance, feeding strategy, and level of bio-security, among other differences. Thus, it is important to investigate whether selection in nucleus farms will in fact lead to a selection response in a commercial production environment. Third, it is unknown whether the additive SGE in purebred animals are equal to those in crossbreds. In Camerlink et al. [7], the pigs were crossbreds, and it is possible that crossbred effects may have nullified any selection differential achieved in SGE based on the purebreds. The variance of SGE may differ between purebreds and crossbreds due to allele frequency differences in the parental breeds and the existence of dominance genetic effects [9]. The genetic correlation between SGE in purebreds and crossbreds may deviate from 1 for the same reasons [10]. In commercial pig production, finishers are crossbreds and, therefore, it is important to verify if social genetic variation for ADG also exists in crossbreds, and that SGE on ADG in purebreds are favourably genetically correlated with SGE on ADG in crossbreds. Fourth, the variation in body weight within groups may be related to social interactions between pigs within groups. Pigs are more aggressive towards each other when the variation in body weight is smaller, probably because weight is related to competitive ability [11], and in weight-matched pigs the establishment and maintenance of dominance relationships is more likely to require fighting. Thus, it is possible that weight-matched pigs will benefit more from SGE on ADG than otherwise, as high SGE pigs may be better at resolving conflicts without aggression [12, 13].

Therefore, the aim of this study was to quantify if selection for SGE on ADG in purebred pigs that are housed in a nucleus farm environment can improve ADG in crossbred pigs in a commercial production environment. For this purpose, the following hypotheses were tested: (1) SGE estimated in purebred pigs have a positive effect on phenotypic ADG in crossbred offspring as reflected by a positive regression coefficient of ADG in crossbred pigs on parent average SGE; (2) expression of SGE for ADG varies with the standard deviation (SD) of starting weight within the group; (3) social genetic variance for ADG exists in crossbred pigs; and (4) a favourable genetic correlation exists between SGE on ADG in purebred and crossbred pigs.

## Methods

### Experimental design and divergent selection on social genetic effects

A large divergent single-generation selection experiment was conducted to investigate the effect of selection for SGE on phenotypic ADG in two-way crossbred, castrated pigs. Production of pigs for the selection experiment was conducted at a single nucleus herd with purebred DanBred Landrace (${\text{L}}$) sows producing F1 crossbred (${\text{YL}}$) piglets with purebred DanBred Yorkshire (${\text{Y}}$) AI boars during the period from October 2016 until August 2018. In total, 199 ${\text{Y}}$-boars and 911 ${\text{L}}$-sows were selected with either high or low SGE for ADG to produce 1171 ${\text{YL}}$ litters. Litters with high SGE were produced by mating boars and sows with high SGE, and litters with low SGE were produced by mating boars and sows with low SGE. On average, 5.3 boars (ranging from 1 to 9) and 13.1 sows (ranging from 7 to 20) were represented within each batch.

The experiment was designed as a split-plot with SGE in main plots, high (> population mean) and low SGE (< population mean) and DGE in sub plots, high (> population mean) and low DGE (< population mean). Therefore, in total there were four combinations of high and low SGE and high and low DGE. For 72 weeks (batches), the offspring of divergently selected purebreds (selection described below) were assigned to main and sub plots based on the average SGE and DGE of the parents at the time of farrowing (selection described below). Pigs were assigned to main plots in the weaner unit and sub plots in the finisher unit. Within each batch, four experimental pens were placed next to each other in the barn in the finisher unit. Pigs were allocated to pens based on the main plot (two pens alongside each other to the left or right) and then split into sub plots. Due to variation in number of litters and litter sizes, the split-plot design was incomplete during 15 of the 72 batches, for which there were only three experimental pens, but there was always at least one pen within each main plot per batch.

DGE and SGE for ADG were estimated weekly within each of the two purebred populations, ${\text{L}}$ and ${\text{Y}}$, in the DanBred breeding program (data described below, and descriptive statistics of the populations at the end of the experiment are in Table 1). The social genetic model used for the selection of both ${\text{L}}$ sows and ${\text{Y}}$ boars was similar to univariate versions of Model 2, which are described later in this paper, with only small differences in covariates, where starting age at the beginning of the performance test was fitted with linear and quadratic terms, along with starting weight (as described in [4], Model 2). In May 2017, the model for ${\text{L}}$ pigs was changed to a bivariate social genetic model in which the sexes of the pigs of boars and gilts were regarded as different traits ([4], Model 4). Then, SGE of the crossbred offspring were calculated as the average SGE of their parents. SGE of crossbreds were on average 1.12 for pigs selected for high SGE and -0.84 for pigs selected for low SGE, resulting in an average selection differential of 1.96 g/day/pig. Whereas the bivariate social genetic model was applied on ${\text{L}}$ pigs, the parent average was based on the average SGE for boars and gilts. After finalizing the experiment, DGE and SGE were re-estimated based on Model 2 (described below) and including all the available purebred and crossbred data (described below). Based on this re-estimation, the SGE of crossbreds were on average 1.36 for pigs with a positive social effect and − 0.24 for pigs with a negative social effect, resulting in an average selection differential of 1.60 g/day/pig.

**Table 1:** Average (standard deviation) performance test statistics for the purebred and crossbred populations

Performance records	Yorkshire*Landrace	Landrace	Yorkshire
Group size (number)	17.4 (1.1)	10.8 (2.2)	11.9 (1.9)
Age at start of test (days)	82.1 (5.3)	80.6 (7.9)	84.8 (8.9)
Age at end of test (days)	130.2 (6.1)	140.2 (16.0)	144.3 (17.2)
Duration of test period (days)	48.2 (2.7)	64.4 (8.6)	65.1 (9.1)
Weight at start of test (kg)	34.0 (6.2)	30.1 (1.9)	29.9 (1.8)
Within-group standard deviation in starting weight	5.2 (1.3)	1.6 (0.4)	1.4 (0.5)
Average daily gain (g/day)	1012 (148)	1018 (128)	1009 (124)

### Production of crossbred pigs and housing

${\text{YL}}$ piglets in the selection experiment were produced by ${\text{L}}$ sows that were housed in individual farrowing crates. Within 24 h after farrowing, all ${\text{YL}}$ piglets were ear-tagged with an individual identification number and coloured ear tags were used to indicate high or low SGE. Semen from sires (${\text{Y}}$) and ${\text{L}}$ sows with high or low SGE were coded by the trial manager with either of two numbers. These numbers were then translated into two colour codes and indicated on the pen work-sheet above the crate of the sow. All staff and technicians were blinded to the meaning of these colour codes. The coloured ear tags were removed in the weaner unit. All male ${\text{YL}}$ piglets were castrated and only the castrates were used in the experiment. Throughout the nursing period, piglets with high SGE were always kept separately from piglets with low SGE, even when cross-fostering or nurse sows were used. Pigs were weaned once per week (referred to as a batch) and transported to a weaner unit at a single commercial finisher herd. The pigs with high or low SGE arrived at the weaner unit on the commercial farm in the same truck, but they were kept separate, and they had ear tags with differing number series. This allowed the commercial farm staff to keep the pigs with high and low SGE separated at all times without knowing which pigs were high and which pigs were low. Pigs with high and low SGE were penned separately, but each batch was housed in a separate section of the barn with all pigs from the same batch housed in the same room. In the weaner unit, the average group size was 40.3 pigs per pen one week following transfer, and ranged from 23 to 45 pigs. Pigs were moved to the finisher unit at an average weight of 34.0 kg at which point the group was divided into two groups of approximately equal size but without introduction of unfamiliar pigs. In the finisher unit, pigs with high and low SGE (main plots) were also housed in separate pens and allocated to groups (different pens) based on high and low DGE (sub plots) as described above. The average group size was 17.4 pigs per pen (ranging from 12 to 19 pigs) and the space allowance per pig was on average 0.82 m^2^/pig (ranging from 0.63 to 1.00 m^2^). If a pig was physically removed from the pen, the removal date and reason were recorded. In total, 4.2% of the pigs did not receive a final weight record due to removal. No pigs were allowed to re-enter their pen after having been removed, and no pigs were allowed to be transferred among experimental pens or from non-experimental pens to experimental pens. Pigs were fed on a standard finisher diet with wet feed three times per day throughout the finisher period. At minimum, the diet fulfilled the Danish standard requirements given by Tybirk et al. [14]. Straw was continuously available in hanging racks, which were filled daily, and pens had 50% solid and 50% slatted floors. Ventilation was semi natural with automatic curtain ventilation on each side of the barn and exhaust fans in the ceiling.

### Recording and crossbred dataset

Individual body weight of the pigs was recorded by two technicians at 24 h and ~ 7 weeks (on average 48.2 days and ranging from 35 to 53 days) after transfer to the finisher unit. Records were collected on a weekly basis, starting from January 2017 until December 2018, including 72 batches, so that each batch received records at two timepoints. All pigs within a batch were weighed on the same two days, and the time interval between first and second weighing, averaging 48.2 days, reflected the test period. The ADG was calculated per individual pig, ${\text{i}}$, as ${\text{ADG}}_{{\text{i}}} = \frac{{{\text{BW}}_{{7{\text{w}},{\text{i}}}} - {\text{ BW}}_{{24{\text{h}},{\text{i}}}} }}{{{\text{DAYS}}_{{{\text{test period}},{\text{i}}}} }}$, where ${\text{BW}}_{{7{\text{w}},{\text{i}}}}$ and ${\text{BW}}_{{24{\text{h}},{\text{i}}}}$ are the body weights at 24 h and ~ 7 weeks after transfer to the finisher unit, and ${\text{DAYS}}_{{{\text{test period}},{\text{ i}}}}$ is the number of days in the test period.

The final crossbred dataset included 4728 individual pigs from 135 groups with high SGE and 138 groups with low SGE, and among these pigs 4464 received a record on ADG. At 24 h after transfer to the finisher unit, pigs with high SGE weighed on average 34.4 kg (SD = 1.2) and were 129.9 days old (SD = 1.5), and pigs with low SGE weighed on average 33.6 kg (SD = 1.1) and were 130.5 days old (SD = 1.8). The calculated ADG during the finisher test period was on average 1015 g/day (SD = 34.0) for pigs with high SGE and 1007 g/day (SD = 38.4) for pigs with low SGE. On an individual level, the crossbred dataset was used to evaluate the effect of SGE on phenotypic ADG (hypothesis a) and the interaction between the sum of the SGE of group mates and the variation in starting weight (hypothesis b) using Model 1 as described below. Descriptive statistics of the crossbred pigs are in Table 1.

### Purebred dataset and pedigree

In order to estimate the genetic variance of SGE in the crossbreds and the genetic correlation between SGE in the purebreds and SGE in the crossbreds (hypotheses 3 and 4), we constructed a dataset with ADG records on performance-tested purebred ${\text{L}}$ and ${\text{Y}}$ boars and gilts in 20 and 22 nucleus herds. The purebred dataset included ${\text{L}}$ (105,265) and ${\text{Y}}$ pigs (148,408) that were performance-tested between November 2013 and February 2018. This dataset included all parents of the pigs in the crossbred dataset along with all performance-tested pigs within the same herd-year-month combinations, which allowed accurate estimation of fixed effect levels of herd-year-month. Purebred pigs were performance-tested for growth until the average body weight within a pen reached 94 kg. In total, 100,160 ${\text{L}}$ pigs and 139,782 ${\text{Y}}$ pigs in 10,184 and 12,828 groups received an ADG record. Average descriptive statistics of the purebred pigs are in Table 1. The average starting weight of purebred ${\text{L}}$ and ${\text{Y}}$ pigs was ~ 30 kg, and at the end of the test, the body weight of all pigs in a given pen was recorded and individual ADG during the test period were calculated. The average ADG was 1018 and 1009 g/day for ${\text{L}}$ and ${\text{Y}}$, respectively. In both breeds, group sizes at the start of the performance test ranged from 7 to 15 pigs per pen. Pen dimensions varied depending on the herd and group size, and the space allowance, and therefore, ranged from 0.75 to 1.0 m^2^/pig. The purebred dataset was used along with the crossbred dataset for the estimation of genetic parameters as described below. Separate pedigrees were traced six generations back for all ${\text{L}}$, ${\text{Y}}$, and ${\text{YL}}$ pigs, and combined into one pedigree which included 111,815 ${\text{L}}$ and 156,476 ${\text{Y}}$ pigs. Based on this pedigree, the average additive genetic relatedness within groups (${\text{r}}$) was 0.153, 0.179, and 0.158 in ${\text{L}}$, ${\text{Y}}$, and ${\text{YL}}$, respectively. Females (gilts) and males (boars) were kept in separate pens and both were fed ad libitum during the test with dry feed, which at a minimum fulfilled the Danish standard requirements [14]. A full description of the housing of ${\text{L}}$ purebreds is in [4], and the housing of ${\text{Y}}$ pigs was similar to that of ${\text{L}}$.

### Effect of SGE on phenotypic ADG

To test the hypothesis that SGE have a positive effect on phenotypic ADG in crossbred pigs, we applied a mixed linear regression (Model 1) to associate the sum of SGE of group mates to individual ADG. We also applied a linear regression on the group level to associate the average group level SGE to the summed ADG per group, which yielded similar results to Model 1 (not reported).

1 $${\mathbf{Y}} \, = \, {\mathbf{F}} + {\text{b}}_{{{\text{DGE}}}} {\mathbf{DGE}} + {\text{b}}_{{{\text{sd}}.{\text{wgt}}}} {\mathbf{sd}}_{{{\mathbf{wgt}}}} + {\text{b}}_{{{\text{cSGE}}}} {\mathbf{cSGE}} + {\text{b}}_{{{\text{wgt}},{\text{SGE}}}} {\mathbf{cSGE}} \times {\mathbf{sd}}_{{{\mathbf{wgt}}}} + {\mathbf{l}} + {\mathbf{e}},$$

where ${\mathbf{Y}}$ is a vector of ADG during the finisher period for the individual ${\text{YL}}$-pigs, ${\mathbf{F}}$ is a vector of individual-based fixed effects including the year of entry in the finisher unit, barn, and regression coefficients for trigonometric functions to account for seasonal effects. The trigonometric functions were calculated as: $\sin \left( {\frac{{{\text{date}}_{{{\text{start}}}} {*}2{\uppi }}}{365}} \right)$ and $\cos (\frac{{{\text{date}}_{{{\text{start}}}} {*}2{\uppi }}}{365})$, where ${\text{date}}_{{{\text{start}}}}$ is the date on which the individual pig entered the finisher unit. The use of sires for the crossbreds was, at least partly, confounded with seasonal variation and the use of sine and cosine functions ensured that only periodic seasonal variation was accounted for in ${\mathbf{F}}$. ${\text{b}}_{{{\text{DGE}}}}$ is the regression coefficient on the vector of parent average ${\mathbf{DGE}}$, ${\text{b}}_{{{\text{sd}}.{\text{wgt}}}}$ is the regression coefficient of the vector on SD of starting weights within group ${\mathbf{sd}}_{{{\mathbf{wgt}}}}$, ${\text{b}}_{{{\text{cSGE}}}}$ is the regression coefficient on the vector of complementary SGE, ${\mathbf{cSGE}}$, i.e. the sum of the group mates’ SGE, calculated as: ${\text{cSGE}}_{{\text{i}}} = \mathop \sum \limits_{{{\text{j}} = 1}}^{{{\text{ng}} - 1}} {\text{SGE}}_{{\text{j}}}$ for each individual pig ${\text{i}}$, ${\text{SGE}}_{{\text{j}}}$ is the parent average SGE of group mate ${\text{j}}$ in group ${\text{g}}$, and ${\text{b}}_{{{\text{wgt}},{\text{SGE}}}}$ is the regression coefficient on the vector of the interaction between ${\mathbf{cSGE}}$ and ${\mathbf{sd}}_{{{\text{wgt}}}}$ to account for differences in the expression of ${\mathbf{cSGE}}_{{\text{i}}}$ depending on ${\mathbf{sd}}_{{{\text{wgt}},{\text{i}}}}$, ${\mathbf{l}}\sim {\text{N}}\left( {0,{\upsigma }_{{\text{l}}}^{2} } \right)$ is the vector of random effects of birth litter and ${\upsigma }_{{\mathbf{l}}}^{2}$ is the variance of the birth litter, and ${\mathbf{e}}\sim {\text{N}}\left( {0,{\upsigma }_{{\text{e}}}^{2} } \right)$ is the vector of random residuals and ${\upsigma }_{{\mathbf{e}}}^{2}$ is the variance of the residuals. Model 1 was analysed with residual maximum likelihood (REML) using the R-package ‘lme4′ [15]. Due to the interaction between ${\mathbf{cSGE}}$ and ${\mathbf{sd}}_{{{\text{wgt}}}}$, the main effect of ${\mathbf{cSGE}}$ at the mean level of ${\mathbf{sd}}_{{{\text{wgt}}}}$ across groups ($\overline{{{\mathbf{sd}}}}_{{{\text{wgt}}}}$ = 5.2 kg) was calculated as: ${\text{b}}_{{{\text{cSGE}}}}^{*} = {\text{b}}_{{{\text{cSGE}}}} + {\text{b}}_{{{\text{wgt}},{\text{SGE}}}} \times \overline{{{\mathbf{sd}}}}_{{{\text{wgt}}}}$. Similarly, the main effect of ${\mathbf{sd}}_{{{\text{wgt}}}}$ at the mean level of ${\mathbf{cSGE}}$ ($\overline{{{\mathbf{cSGE}}}}$ = 8.9) was calculated as: ${\text{b}}_{{{\text{sd}}_{{{\text{wgt}}}} }}^{*} = {\text{b}}_{{{\text{sd}}_{{{\text{wgt}}}} }} + {\text{b}}_{{{\text{wgt}},{\text{SGE}}}} \times \overline{{{\mathbf{cSGE}}}}$.

### Estimation of genetic parameters

A trivariate linear mixed model (Model 2) was used for ADG in all three populations to estimate (co)-variance components for both direct and social genetic effects. Social genetic animal models were described for purebred ${\text{L}}$- and ${\text{Y}}$-pigs and combined with a social genetic sire-dam model described for crossbred ${\text{YL}}$-pigs.

2 $$\begin{aligned} {\mathbf{y}}_{{\mathbf{L}}} = \, & {\mathbf{X}}_{{\mathbf{L}}} {\mathbf{b}}_{{\mathbf{L}}} + {\mathbf{Z}}_{{\mathbf{L}}}^{{\mathbf{D}}} {\mathbf{a}}_{{\mathbf{L}}}^{{\mathbf{D}}} + {\mathbf{Z}}_{{\mathbf{L}}}^{{\mathbf{S}}} {\mathbf{a}}_{{\mathbf{L}}}^{{\mathbf{S}}} + {\mathbf{Z}}_{{{\mathbf{l}},{\mathbf{L}}}} {\mathbf{l}}_{{\mathbf{L}}} + {\mathbf{Z}}_{{{\mathbf{g}},{\mathbf{L}}}} {\mathbf{g}}_{{\mathbf{L}}} + {\mathbf{e}}_{{\mathbf{L}}} ,{ } \\ {\mathbf{y}}_{{\mathbf{Y}}} = \, & {\mathbf{X}}_{{\mathbf{Y}}} {\mathbf{b}}_{{\mathbf{Y}}} + {\mathbf{Z}}_{{\mathbf{Y}}}^{{\mathbf{D}}} {\mathbf{a}}_{{\mathbf{Y}}}^{{\mathbf{D}}} + {\mathbf{Z}}_{{\mathbf{Y}}}^{{\mathbf{S}}} {\mathbf{a}}_{{\mathbf{Y}}}^{{\mathbf{S}}} + {\mathbf{Z}}_{{{\mathbf{l}},{\mathbf{Y}}}} {\mathbf{l}}_{{\mathbf{Y}}} + {\mathbf{Z}}_{{{\mathbf{g}},{\mathbf{Y}}}} {\mathbf{g}}_{{\mathbf{Y}}} + {\mathbf{e}}_{{\mathbf{Y}}} , \\ {\mathbf{y}}_{{{\mathbf{YL}}}} = \, & {\mathbf{X}}_{{{\mathbf{YL}}}} {\mathbf{b}}_{{{\mathbf{YL}}}} + {\mathbf{Z}}_{{{\mathbf{L}} - {\mathbf{YL}}}}^{{\mathbf{D}}} {\mathbf{a}}_{{{\mathbf{L}} - {\mathbf{YL}}}}^{{\mathbf{D}}} + {\mathbf{Z}}_{{{\mathbf{Y}} - {\mathbf{YL}}}}^{{\mathbf{D}}} {\mathbf{a}}_{{{\mathbf{Y}} - {\mathbf{YL}}}}^{{\mathbf{D}}} + {\mathbf{Z}}_{{{\mathbf{L}} - {\mathbf{YL}}}}^{{\mathbf{S}}} {\mathbf{a}}_{{{\mathbf{L}} - {\mathbf{YL}}}}^{{\mathbf{S}}} + {\mathbf{Z}}_{{{\mathbf{Y}} - {\mathbf{YL}}}}^{{\mathbf{S}}} {\mathbf{a}}_{{{\mathbf{Y}} - {\mathbf{YL}}}}^{{\mathbf{S}}} + {\mathbf{Z}}_{{{\mathbf{l}},{\mathbf{YL}}}} {\mathbf{l}}_{{{\mathbf{YL}}}} + {\mathbf{Z}}_{{{\mathbf{g}},{\mathbf{YL}}}} {\mathbf{g}}_{{{\mathbf{YL}}}} + {\mathbf{e}}_{{{\mathbf{YL}}}}, \\ \end{aligned}$$

where ${\mathbf{y}}_{{\mathbf{L}}}$, ${\mathbf{y}}_{{\mathbf{Y}}}$, and ${\mathbf{y}}_{{{\mathbf{YL}}}}$ are vectors of the ADG records with subscripts ${\text{L}}$, ${\text{Y}}$, and ${\text{YL}}$ denoting the purebred, ${\text{L}}$ and ${\text{Y}}$, and crossbred, ${\text{YL}}$, pigs. The vectors of covariates and fixed effects (${\mathbf{b}}_{{\mathbf{L}}}$, ${\mathbf{b}}_{{\mathbf{Y}}}$, and ${\mathbf{b}}_{{{\mathbf{YL}}}}$) are given per population with their associated block incidence matrices, ${\mathbf{X}}_{{\mathbf{L}}}$, ${\mathbf{X}}_{{\mathbf{Y}}}$, and ${\mathbf{X}}_{{{\mathbf{YL}}}}$. For the purebreds, the fixed effects included sex (boar or gilt), contemporary batch, defined as pigs reared and performance-tested within the same farm and time period (final performance test date within approximately one month), and starting weight as a covariate. For the crossbreds, fixed effects and covariates were as described for Model 1. ${\mathbf{a}}_{{\mathbf{L}}}^{{\mathbf{D}}}$, ${\mathbf{a}}_{{\mathbf{Y}}}^{{\mathbf{D}}}$, and ${\mathbf{a}}_{{{\mathbf{YL}}}}^{{\mathbf{D}}}$ are the vectors of random direct genetic effects, and ${\mathbf{a}}_{{\mathbf{L}}}^{{\mathbf{S}}}$, ${\mathbf{a}}_{{\mathbf{Y}}}^{{\mathbf{S}}}$, and ${\mathbf{a}}_{{{\mathbf{YL}}}}^{{\mathbf{S}}}$ are the vectors of random social genetic effects. The incidence matrices ${\mathbf{Z}}_{{\mathbf{L}}}^{{\mathbf{D}}}$, ${\mathbf{Z}}_{{\mathbf{L}}}^{{\mathbf{S}}}$, ${\mathbf{Z}}_{{\mathbf{Y}}}^{{\mathbf{D}}}$, and ${\mathbf{Z}}_{{\mathbf{Y}}}^{{\mathbf{S}}}$ link the records on the purebreds to direct and social genetic effects in the purebreds. Similarly, ${\mathbf{Z}}_{{{\mathbf{L}} - {\mathbf{YL}}}}^{{\mathbf{D}}}$, ${\mathbf{Z}}_{{{\mathbf{L}} - {\mathbf{YL}}}}^{{\mathbf{S}}}$, ${\mathbf{Z}}_{{{\mathbf{Y}} - {\mathbf{YL}}}}^{{\mathbf{D}}}$, and ${\mathbf{Z}}_{{{\mathbf{Y}} - {\mathbf{YL}}}}^{{\mathbf{S}}}$ link the records on the crossbreds to DGE and SGE in the purebreds. Non-zero elements of ${\mathbf{Z}}_{{{\mathbf{L}} - {\mathbf{YL}}}}^{{\mathbf{D}}}$, ${\mathbf{Z}}_{{{\mathbf{L}} - {\mathbf{YL}}}}^{{\mathbf{S}}}$, ${\mathbf{Z}}_{{{\mathbf{Y}} - {\mathbf{YL}}}}^{{\mathbf{D}}}$, and ${\mathbf{Z}}_{{{\mathbf{Y}} - {\mathbf{YL}}}}^{{\mathbf{S}}}$ are 0.5, as only half of the genes are passed on from each purebred parent to its crossbred progeny. ${\mathbf{l}}_{{\mathbf{L}}}$, ${\mathbf{l}}_{{\mathbf{Y}}}$, and ${\mathbf{l}}_{{{\mathbf{YL}}}}$ are the vectors of random litter effects, and ${\mathbf{g}}_{{\mathbf{L}}}$, ${\mathbf{g}}_{{\mathbf{Y}}}$, and ${\mathbf{g}}_{{{\mathbf{YL}}}}$ are the vectors of random group effects. The incidence matrices ${\mathbf{Z}}_{{{\mathbf{l}},{\mathbf{L}}}}$, ${\mathbf{Z}}_{{{\mathbf{l}},{\mathbf{Y}}}}$, and ${\mathbf{Z}}_{{{\mathbf{l}},{\mathbf{YL}}}}$ link the records to the random litter effects, and incidence matrices ${\mathbf{Z}}_{{{\mathbf{g}},{\mathbf{L}}}}$, ${\mathbf{Z}}_{{{\mathbf{g}},{\mathbf{Y}}}}$, and ${\mathbf{Z}}_{{{\mathbf{g}},{\mathbf{YL}}}}$ link the records to the random group effects. ${\mathbf{e}}_{{\mathbf{L}}}$, ${\mathbf{e}}_{{\mathbf{Y}}}$, and ${\mathbf{e}}_{{{\mathbf{YL}}}}$ are the vectors of residuals. Direct and social genetic effects are assumed to be jointly normally distributed:

$$\left( {\begin{array}{*{20}c} {{\mathbf{a}}_{{\mathbf{L}}}^{{\mathbf{D}}} } \\ {{\mathbf{a}}_{{\mathbf{L}}}^{{\mathbf{S}}} } \\ {{\mathbf{a}}_{{{\mathbf{L}} - {\mathbf{YL}}}}^{{\mathbf{D}}} } \\ {{\mathbf{a}}_{{{\mathbf{L}} - {\mathbf{YL}}}}^{{\mathbf{S}}} } \\ {{\mathbf{a}}_{{\mathbf{Y}}}^{{\mathbf{D}}} } \\ {{\mathbf{a}}_{{\mathbf{Y}}}^{{\mathbf{S}}} } \\ {{\mathbf{a}}_{{{\mathbf{Y}} - {\mathbf{YL}}}}^{{\mathbf{D}}} } \\ {{\mathbf{a}}_{{{\mathbf{Y}} - {\mathbf{YL}}}}^{{\mathbf{S}}} } \\ \end{array} } \right){ }\sim {\text{N}}\left( {0,\left[ {\begin{array}{*{20}c} {{\mathbf{G}}_{{\mathbf{L}}} \otimes {\mathbf{A}}_{{\mathbf{L}}} } & 0 \\ 0 & {{\mathbf{G}}_{{\mathbf{Y}}} \otimes {\mathbf{A}}_{{\mathbf{Y}}} } \\ \end{array} } \right]} \right),$$

where ${\mathbf{A}}_{{\mathbf{L}}}$ and ${\mathbf{A}}_{{\mathbf{Y}}}$ are the additive genetic relationship matrices for purebred ${\text{L}}$ and ${\text{Y}}$ populations, respectively, $\otimes$ is the Kronecker product, and ${\mathbf{G}}_{{\mathbf{L}}}$ and ${\mathbf{G}}_{{\mathbf{Y}}}$ are (co)variance matrices:

$${\mathbf{G}}_{{\mathbf{L}}} = \left[ {\begin{array}{*{20}c} {{\upsigma }_{{{\text{D}},{\text{L}}}}^{2} } & {} & {\begin{array}{*{20}c} {} & {} \\ \end{array} } \\ {{\upsigma }_{{{\text{DS}},{\text{L}}}} } & {{\upsigma }_{{{\text{S}},{\text{L}}}}^{2} } & {\begin{array}{*{20}c} {} & {} \\ \end{array} } \\ {\begin{array}{*{20}c} {{\upsigma }_{{{\text{D}},{\text{L}};{\text{D}},{\text{L}} - {\text{YL}}}} } \\ {{\upsigma }_{{{\text{D}},{\text{L}};{\text{S}},{\text{L}} - {\text{YL}}}} } \\ \end{array} } & {\begin{array}{*{20}c} {{\upsigma }_{{{\text{S}},{\text{L}};{\text{D}},{\text{L}} - {\text{YL}}}} } \\ {{\upsigma }_{{{\text{S}},{\text{L}};{\text{S}},{\text{L}} - {\text{YL}}}} } \\ \end{array} } & {\begin{array}{*{20}c} {\begin{array}{*{20}c} {{\upsigma }_{{{\text{D}},{\text{L}} - {\text{YL}}}}^{2} } & {} \\ \end{array} } \\ {\begin{array}{*{20}c} {{\upsigma }_{{{\text{DS}},{\text{L}} - {\text{YL}}}} } & {{\upsigma }_{{{\text{S}},{\text{L}} - {\text{YL}}}}^{2} } \\ \end{array} } \\ \end{array} } \\ \end{array} } \right]{ ,}$$

$${\mathbf{G}}_{{\mathbf{Y}}} = \left[ {\begin{array}{*{20}c} {{\upsigma }_{{{\text{D}},{\text{Y}}}}^{2} } & {} & {\begin{array}{*{20}c} {} & {} \\ \end{array} } \\ {{\upsigma }_{{{\text{DS}},{\text{Y}}}} } & {{\upsigma }_{{{\text{S}},{\text{Y}}}}^{2} } & {\begin{array}{*{20}c} {} & {} \\ \end{array} } \\ {\begin{array}{*{20}c} {{\upsigma }_{{{\text{D}},{\text{Y}};{\text{D}},{\text{Y}} - {\text{YL}}}} } \\ {{\upsigma }_{{{\text{D}},{\text{Y}};{\text{S}},{\text{Y}} - {\text{YL}}}} } \\ \end{array} } & {\begin{array}{*{20}c} {{\upsigma }_{{{\text{S}},{\text{Y}};{\text{D}},{\text{Y}} - {\text{YL}}}} } \\ {{\upsigma }_{{{\text{S}},{\text{Y}};{\text{S}},{\text{Y}} - {\text{YL}}}} } \\ \end{array} } & {\begin{array}{*{20}c} {\begin{array}{*{20}c} {{\upsigma }_{{{\text{D}},{\text{Y}} - {\text{YL}}}}^{2} } & {} \\ \end{array} } \\ {\begin{array}{*{20}c} {{\upsigma }_{{{\text{DS}},{\text{Y}} - {\text{YL}}}} } & {{\upsigma }_{{{\text{S}},{\text{Y}} - {\text{YL}}}}^{2} } \\ \end{array} } \\ \end{array} } \\ \end{array} } \right].$$

The diagonal elements in ${\mathbf{G}}_{{\mathbf{L}}}$ represent the genetic variances of direct social genetic effects in ${\text{L}}$-pigs and in the dam component (${\text{L}}$) of crossbred pigs. The off-diagonal elements describe the genetic covariances among direct and social genetic effects in ${\text{L}}$ pigs and the dam component (${\text{L}}$) of crossbred pigs. Similarly, the elements in ${\mathbf{G}}_{{\mathbf{Y}}}$ represent the genetic variances and covariances in ${\text{Y}}$ pigs and the sire component (${\text{Y}}$) of crossbred pigs. Random litter (${\mathbf{l}}_{{\mathbf{L}}}$, ${\mathbf{l}}_{{\mathbf{Y}}}$, and ${\mathbf{l}}_{{{\mathbf{YL}}}}$) and group effects (${\mathbf{g}}_{{\mathbf{L}}}$, ${\mathbf{g}}_{{\mathbf{Y}}}$, and ${\mathbf{g}}_{{{\mathbf{YL}}}}$) for ${\text{L}}$, ${\text{Y}}$ and ${\text{YL}}$ pigs were assumed to be independent and normally distributed: ${\mathbf{l}}_{{\mathbf{L}}} \sim {\text{N}}\left( {0,{\mathbf{I}}_{{{\mathbf{l}},{\mathbf{L}}}} {\upsigma }_{{{\text{l}}_{{\text{L}}} }}^{2} } \right)$, ${\mathbf{l}}_{{\mathbf{Y}}} \sim {\text{N}}\left( {0,{\mathbf{I}}_{{{\mathbf{l}},{\mathbf{Y}}}} {\upsigma }_{{{\text{l}}_{{\text{Y}}} }}^{2} } \right)$, ${\mathbf{l}}_{{{\mathbf{YL}}}} \sim {\text{N}}\left( {0,{\mathbf{I}}_{{{\mathbf{l}},{\mathbf{YL}}}} {\upsigma }_{{{\text{l}}_{{{\text{YL}}}} }}^{2} } \right)$, ${\mathbf{g}}_{{\mathbf{L}}} \sim {\text{N}}\left( {0,{\mathbf{I}}_{{{\mathbf{g}},{\mathbf{L}}}} {\upsigma }_{{{\text{g}}_{{\text{L}}} }}^{2} } \right)$, ${\mathbf{g}}_{{\mathbf{Y}}} \sim {\text{N}}\left( {0,{\mathbf{I}}_{{{\mathbf{g}},{\mathbf{Y}}}} {\upsigma }_{{{\text{g}}_{{\text{Y}}} }}^{2} } \right)$, and ${\mathbf{g}}_{{{\mathbf{YL}}}} \sim {\text{N}}\left( {0,{\mathbf{I}}_{{{\mathbf{g}},{\mathbf{YL}}}} {\upsigma }_{{{\text{g}}_{{{\text{YL}}}} }}^{2} } \right)$. Likewise, the residual effects (${\mathbf{e}}_{{\mathbf{L}}}$, ${\mathbf{e}}_{{\mathbf{Y}}}$, and ${\mathbf{e}}_{{{\mathbf{YL}}}}$) for ${\text{L}}$, ${\text{Y}}$, and ${\text{YL}}$ pigs were assumed to be uncorrelated and normally distributed: ${\mathbf{e}}_{{\mathbf{L}}} \sim {\text{N}}\left( {0,{\mathbf{I}}_{{{\mathbf{e}},{\mathbf{L}}}} {\upsigma }_{{{\text{e}}_{{\text{L}}} }}^{2} } \right)$, ${\mathbf{e}}_{{\mathbf{Y}}} \sim {\text{N}}\left( {0,{\mathbf{I}}_{{{\mathbf{e}},{\mathbf{Y}}}} {\upsigma }_{{{\text{e}}_{{\text{Y}}} }}^{2} } \right)$, and ${\mathbf{e}}_{{{\mathbf{YL}}}} \sim {\text{N}}\left( {0,{\mathbf{I}}_{{{\mathbf{e}},{\mathbf{YL}}}} {\upsigma }_{{{\text{e}}_{{{\text{YL}}}} }}^{2} } \right)$. The identity matrices ${\mathbf{I}}_{{{\mathbf{l}},{\mathbf{L}}}}$, ${\mathbf{I}}_{{{\mathbf{l}},{\mathbf{Y}}}}$, ${\mathbf{I}}_{{{\mathbf{l}},{\mathbf{YL}}}}$, ${\mathbf{I}}_{{{\mathbf{g}},{\mathbf{L}}}}$, ${\mathbf{I}}_{{{\mathbf{g}},{\mathbf{Y}}}}$, ${\mathbf{I}}_{{{\mathbf{g}},{\mathbf{YL}}}}$, ${\mathbf{I}}_{{{\mathbf{e}},{\mathbf{L}}}}$, ${\mathbf{I}}_{{{\mathbf{e}},{\mathbf{Y}}}}$, and ${\mathbf{I}}_{{{\mathbf{e}},{\mathbf{YL}}}}$ are of dimensions equal to the numbers of litters, groups, and observations on ADG on ${\text{L}}$, ${\text{Y}}$, and ${\text{YL}}$ pigs, respectively.

Parameters including the genetic (co-)variances were estimated using average information REML (AI-REML) using the DMU software [16] release 5.3. Based on (co)-variances from Model 2, the following parameters were calculated. Approximated phenotypic variances ($\sigma_{P,k}^{2}$) in purebreds $k = \left\{ {L,Y} \right\}$ were calculated for related individuals as [17]: ${\upsigma }_{{{\text{P}},{\text{k}}}}^{2} = {\upsigma }_{{{\text{D}},{\text{k}}}}^{2} + \left( {n - 1} \right){\upsigma }_{{{\text{S}},{\text{k}}}}^{2} + r\left( {n - 1} \right)\left[ {2{\upsigma }_{{{\text{DS}},{\text{k}}}} + \left( {n - 2} \right){\upsigma }_{{{\text{S}},{\text{k}}}}^{2} } \right] + {{ \sigma }}_{{{\text{l}},{\text{k}}}}^{2} + {\upsigma }_{{{\text{g}},{\text{k}}}}^{2} + {{ \sigma }}_{{{\text{e}},{\text{k}}}}^{2}$, where $r$ is the average additive genetic relationship within groups and $n$ is the average group size at the start of the performance test. The approximated total genetic variances, $\sigma_{TGE,k}^{2}$, available for selection in the purebreds for response in the crossbreds, $k = \left\{ {L - YL, Y - YL} \right\}$ were calculated as [18]: ${\upsigma }_{{{\text{TGE}},{\text{k}}}}^{2} = {{ \sigma }}_{{{\text{D}},{\text{k}}}}^{2} + 2\left( {n - 1} \right){\upsigma }_{{{\text{DS}},{\text{k}}}} + \left( {n - 1} \right)^{2} {\upsigma }_{{{\text{S}},{\text{k}}}}^{2}$, where ${\text{TGE}}$ is the total genetic effect, which is the sum of ${\text{DGE}}$ and ${\text{cSGE}}$, i.e. the sum of SGE of the group mates. The approximated standard error (SE) of $\sigma_{{{\text{TGE}}}}^{2}$ was calculated as: ${\text{SE}}_{{{\hat{\sigma }}_{{{\text{TGE}}}}^{2} }} = \frac{1}{r}\sqrt {\frac{2}{{{\text{N}} - 1}}\left[ {{\upsigma }_{{\text{f}}}^{4} + \frac{{2{\upsigma }_{{\text{f}}}^{2} {\upsigma }_{{\text{e}}}^{2} }}{m} + \frac{{{\upsigma }_{{\text{e}}}^{4} }}{{m\left( {m - 1} \right)}}} \right]}$, where ${\upsigma }_{{\text{f}}}^{2} = r{\upsigma }_{{{\text{TGE}}}}^{2}$ is the between-family variance, ${\text{N}}$ is the number of families (litters in the data), and $m$ is the family size (calculated as the total number of individuals in the data divided by the number of families/litters in the data) [19]. The direct heritability for purebreds, ${\text{L}}$ and ${\text{Y}}$, was calculated as: ${\text{h}}^{2} = \frac{{{\upsigma }_{{\text{D}}}^{2} }}{{{\upsigma }_{{\text{P}}}^{2} }}$, and the SE of ${\text{h}}^{2}$ was calculated as: ${\text{SE}}_{{{\hat{\text{h}}}_{{\text{D}}}^{2} }} = {\text{SE}}_{{{\hat{\sigma }}_{{{\text{A}}_{{\text{D}}} }}^{2} }} /{\upsigma }_{{\text{P}}}^{2}$ [19]. Since selection is performed in the purebreds and genetic parameters reflect genetic variation present in the purebreds, the phenotypic and total genetic variance and the heritabilities were calculated in the purebreds only.

### Test of genetic covariances between SGE in purebreds and crossbreds

To test whether there was a significant relationship between SGE in purebreds and crossbreds, we used a likelihood-ratio test to compare if the full genetic model (Model 2) had a better fit than a reduced model (Model 3). The reduced model was equivalent to Model 2, except that covariances including SGE between purebred and crossbred performance were fixed to 0 in the genetic (co)variance matrices, i.e.:

3 $$\begin{gathered} {\mathbf{G}}_{{\mathbf{L}}} = \left[ {\begin{array}{*{20}c} {{\upsigma }_{{{\text{D}},{\text{L}}}}^{2} } & {} & {\begin{array}{*{20}c} {} & {} \\ \end{array} } \\ {{\upsigma }_{{{\text{DS}},{\text{L}}}} } & {{\upsigma }_{{{\text{S}},{\text{L}}}}^{2} } & {\begin{array}{*{20}c} {} & {} \\ \end{array} } \\ {\begin{array}{*{20}c} {{\upsigma }_{{{\text{D}},{\text{L}};{\text{D}},{\text{L}} - {\text{YL}}}} } \\ 0 \\ \end{array} } & {\begin{array}{*{20}c} 0 \\ 0 \\ \end{array} } & {\begin{array}{*{20}c} {\begin{array}{*{20}c} {{\upsigma }_{{{\text{D}},{\text{L}} - {\text{YL}}}}^{2} } & {} \\ \end{array} } \\ {\begin{array}{*{20}c} {{\upsigma }_{{{\text{DS}},{\text{L}} - {\text{YL}}}} } & {{\upsigma }_{{{\text{S}},{\text{L}} - {\text{YL}}}}^{2} } \\ \end{array} } \\ \end{array} } \\ \end{array} } \right]{ } \hfill \\ {\mathbf{G}}_{{\mathbf{Y}}} = \left[ {\begin{array}{*{20}c} {{\upsigma }_{{{\text{D}},{\text{Y}}}}^{2} } & {} & {\begin{array}{*{20}c} {} & {} \\ \end{array} } \\ {{\upsigma }_{{{\text{DS}},{\text{Y}}}} } & {{\upsigma }_{{{\text{S}},{\text{Y}}}}^{2} } & {\begin{array}{*{20}c} {} & {} \\ \end{array} } \\ {\begin{array}{*{20}c} {{\upsigma }_{{{\text{D}},{\text{Y}};{\text{D}},{\text{Y}} - {\text{YL}}}} } \\ 0 \\ \end{array} } & {\begin{array}{*{20}c} 0 \\ 0 \\ \end{array} } & {\begin{array}{*{20}c} {\begin{array}{*{20}c} {{\upsigma }_{{{\text{D}},{\text{Y}} - {\text{YL}}}}^{2} } & {} \\ \end{array} } \\ {\begin{array}{*{20}c} {{\upsigma }_{{{\text{DS}},{\text{Y}} - {\text{YL}}}} } & {{\upsigma }_{{{\text{S}},{\text{Y}} - {\text{YL}}}}^{2} } \\ \end{array} } \\ \end{array} } \\ \end{array} } \right], \hfill \\ \end{gathered}$$

The likelihood-ratio test was: ${\text{P}}\left( {\Delta {\text{logL}}} \right) = {\upchi }^{2} \left( { - 2{\text{log}}_{{\text{e}}} \left[ {\frac{{\ell \left( {{\text{reduced}}} \right)}}{{\ell \left( {{\text{full}}} \right)}}} \right],{\text{df}}} \right)$, where ${\upchi }^{2} \left( \ldots \right)$ was the cumulative distribution function of the chi-square distribution, $\ell \left( {{\text{reduced}}} \right)$ was the likelihood value of the reduced model (Model 3), and $\ell \left( {{\text{full}}} \right)$ was the likelihood value of Model 2 with df = 6.

## Results

### Effect of SGE on phenotypic ADG

**Fig. 1:**
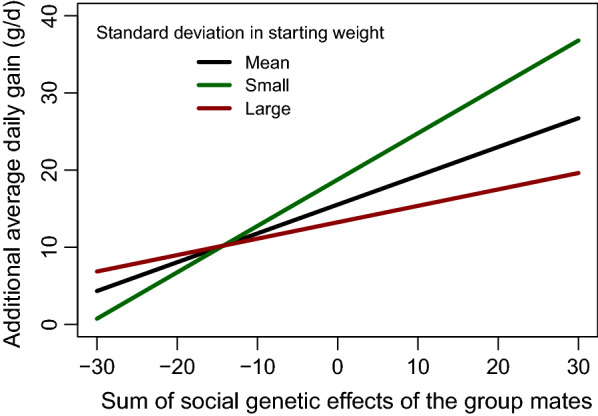
Effect of the sum of SGE of group mates on average daily gain (ADG) depending on variation in starting weight within the group, where ‘small’ and ‘large’ correspond to the 1st and 3rd quartiles, respectively

Table 2 shows the estimated regression coefficients and variances from Model 1. There was a negative and statistically significant regression coefficient (-0.26) for the interaction between the SD in starting weight and ${\text{cSGE}}$ on ADG (${\text{b}}_{{{\text{sd}}.{\text{wgt}},{\text{SGE}}}}$). Conditioning on the mean SD in starting weight across groups, the regression of ADG in crossbreds on SGE in purebreds was ${\text{b}}_{{{\text{cSGE}}}}^{*}$ = 0.37 ± 0.21 g/day. This means, that within a given group, the ADG of each pig will increase by 0.37 g/day, when the sum of its group mates’ SGE increases by 1 g/day. The regression coefficient of DGE (${\text{b}}_{{{\text{DGE}}}}$) was statistically significant and favourable (0.30). Conditioning on the mean ${\text{cSGE}}$ across groups, the regression coefficient of the SD of starting weight (${\text{b}}_{{{\text{sd}}.{\text{wgt}}}}^{*}$) was negative (− 6.09 ± 2.00). Thus, the highest ADG per pig (and group) is achieved with a small SD in starting weight and a highly favourable average ${\text{cSGE}}$ of the group. This is illustrated in Fig. 1 with three levels of SD in starting weight: small (1st quartile), mean, and large (3rd quartile) values. The levels (1st quartile, mean, 3rd quartile) of SD in starting weight in the crossbreds were: 4.3, 5.2, and 5.8, and the levels (1st quartile, mean, 3rd quartile) of cSGE of the crossbred groups were: − 4.0, 8.9, and 22.1.

**Table 2:** Regression coefficients and variance estimates with associated standard errors and p-values based on average daily gain (g/day) in crossbreds (Model 1)

	Estimate	p-value
${\text{b}}_{{{\text{cSGE}}}}^{*}$	0.37 (0.21)	
${\text{b}}_{{{\text{DGE}}}}$	0.30 (0.12)	0.02*
${\text{b}}_{{{\text{sd}}.{\text{wgt}}}}^{*}$	-6.09 (2.00)	
${\text{b}}_{{{\text{sd}}.{\text{wgt}},{\text{SGE}}}}$	-0.26 (0.12)	0.03*
${\upsigma }_{{\text{l}}}^{2}$	3060 (55)	
${\upsigma }_{{\text{e}}}^{2}$	16,864 (130)	

${\text{b}}_{{{\text{cSGE}}}}^{*}$: regression coefficient of complementary social genetic effect, ${\text{cSGE}}$, i.e. the sum of the SGE of group mates, conditional on the mean standard deviation in starting weight across groups; ${\text{b}}_{{{\text{DGE}}}}$: regression coefficient of the direct genetic effect, DGE; ${\text{b}}_{{{\text{sd}}.{\text{wgt}}}}$: regression coefficient of the standard deviation of starting weight within group; ${\text{b}}_{{{\text{sd}}.{\text{wgt}},{\text{SGE}}}}$: regression coefficient of the interaction between the standard deviation of starting weight within group and cSGE; ${\upsigma }_{{\text{l}}}^{2}$ and ${\upsigma }_{{\text{e}}}^{2}$: the estimated variances of random birth litter effects and residual effects. Statistical significance for estimates differing from zero is indicated by stars (*P < 0.05)

### Genetic parameters in purebreds and crossbreds

The estimated genetic (co-)variances and genetic correlations for ${\text{L}}$ and the dam component of ${\text{YL}}$ are in Table 3 and those for ${\text{Y}}$ and the sire component of ${\text{YL}}$ are in Table 4.

**Table 3:** Direct and social genetic variances for average daily gain (g/day) on the diagonal, genetic covariances below the diagonal, and genetic correlations above the diagonal from matrix ${\text{G}}_{{\text{L}}}$ in Model 2 for Landrace (${\text{L}}$) and the ${\text{L}}$ dam component in the crossbreds (${\text{YL}}$)

	Genetic effect	${\mathbf{L}}$		Dam (${\mathbf{YL}}$)	
		Direct	Social	Direct	Social
${\text{L}}$	Direct	2659 (135)	0.00 (0.11)	0.46 (0.18)	− 0.09 (0.22)
	Social	0.3 (13.8)	5.9 (2.0)	0.66 (0.36)	0.52 (0.48)
Dam (${\text{YL}}$)	Direct	1216 (469)	82.0 (47.3)	2608 (1125)	− 0.18 (0.38)
	Social	− 30.2 (72.8)	8.2 (7.7)	− 60.5 (128)	41.5 (28.2)

Standard errors are shown in brackets

**Table 4:** Direct and social genetic variances for average daily gain (g/day) on the diagonal, genetic covariances below the diagonal, and genetic correlations above the diagonal from matrix ${\text{G}}_{{\text{Y}}}$ in Model 2 for Yorkshire (${\text{Y}}$) and the Y sire component in the crossbreds (${\text{YL}}$)S

	Genetic effect	${\mathbf{Y}}$		Sire (${\mathbf{YL}}$)	
		Direct	Social	Direct	Social
${\text{Y}}$	Direct	3150 (129)	0.07 (0.10)	0.41 (0.17)	− 0.22 (0.26)
	Social	8.8 (13.6)	5.3 (1.7)	− 0.75 (0.28)	0.34 (0.42)
Sire (${\text{YL}}$)	Direct	1669 (720)	− 124 (52)	5187 (1378)	− 0.48 (0.22)
	Social	− 64 (76)	4.1 (5.1)	− 181 (109)	27.5 (14.3)

Standard errors are shown in brackets

Estimates of variances of DGE in ${\text{L}}$, ${\text{Y}}$, and the dam component of ${\text{YL}}$ were of similar magnitude (between 2608 and 3150), whereas the variance of the sire component of ${\text{YL}}$ was almost twice the size of the variance of the dam component of ${\text{YL}}$ (5187). There were small, but significant variances of SGE in both ${\text{L}}$ and ${\text{Y}}$ (5.9 and 5.3, respectively). The variances of SGE in both the dam and sire component of ${\text{YL}}$ were higher (41.5 and 27.5, respectively), but none were statistically significant.

The genetic correlations between DGE in the purebreds (${\text{L}}$ or ${\text{Y}}$) and the dam or sire component of the crossbreds (${\text{YL}}$) were both statistically significant and favourable (0.46 and 0.41, respectively). The genetic correlations between SGE in the purebreds (${\text{L}}$ or ${\text{Y}}$) and the dam or sire component of the crossbreds were also favourable (0.52 and 0.34, respectively), although not statistically significant. The genetic correlations between DGE and SGE within both the purebred populations were 0 or close to 0 and not statistically significant from 0. In contrast, the corresponding correlations within the crossbreds for both the dam (${\text{L}}$) and the sire (${\text{Y}}$) component were both unfavourable (− 0.18 and − 0.48, respectively), although also not statistically significantly different from 0.

Table 5 includes the non-genetic variances, heritabilities and total genetic variances for the purebreds, ${\text{L}}$ and ${\text{Y}}$. The random group and residual variances were higher in ${\text{Y}}$ than ${\text{L}}$, whereas the random litter variance was higher in ${\text{L}}$ than ${\text{Y}}$. Direct heritabilities were similar in L and ${\text{Y}}$ (0.25 and 0.27). The total genetic variance available for selection in ${\text{L}}$ or ${\text{Y}}$ for response in the crossbreds was higher than the total genetic variance available for response in the purebreds. In ${\text{L}}$, the total genetic variance available for selection to yield response in the crossbreds (5408) was also higher than the corresponding direct genetic variance (2608, see Table 3). However, in ${\text{Y}}$ the total genetic variance available for selection to yield response in the crossbreds (4508) was lower than the corresponding direct genetic variance (5187, see Table 4).

**Table 5:** Variances^a^ of random group- (${\upsigma }_{{\text{g}}}^{2}$), litter- (${\upsigma }_{{\text{l}}}^{2}$), and residual (${\upsigma }_{{\text{e}}}^{2}$) effects, phenotypic (${\upsigma }_{{\text{P}}}^{2}$) and total genetic variance (${\upsigma }_{{{\text{TGE}}}}^{2}$), and direct heritability (${\text{h}}^{2}$) for average daily gain (g/day) in Landrace (${\text{L}}$), Yorkshire (${\text{Y}}$), and crossbreds (${\text{YL}}$)

Population	${{\varvec{\upsigma}}}_{{\mathbf{g}}}^{2}$	${{\varvec{\upsigma}}}_{{\mathbf{l}}}^{2}$	${{\varvec{\upsigma}}}_{{\mathbf{e}}}^{2}$	${{\varvec{\upsigma}}}_{{\mathbf{P}}}^{2}$	${{\varvec{\upsigma}}}_{{{\mathbf{TGE}}}}^{2}$	${\mathbf{h}}^{2}$	${{\varvec{\upsigma}}}_{{{\mathbf{TGE}},{\mathbf{k}} - {\mathbf{YL}}}}^{2}$ ^a^
${\text{L}}$	777 (33)	654 (29)	6373 (72)	10,588 (93)	3167 (336)	0.25 (0.01)	5408 (785)
${\text{Y}}$	939 (33)	539 (24)	6977 (68)	11,780 (97)	3972 (403)	0.27 (0.01)	4508 (799)

Standard errors are shown in brackets

^a^${\upsigma }_{{{\text{TGE}},{\text{k}} - {\text{YL}}}}^{2}$ is the total genetic variance available for selection in the purebreds to obtain genetic response in the crossbreds, YL, where $k = \left\{ {L,Y} \right\}$

The log-likelihood test of the reduced Model 3 against the full Model 2 showed a non-significant effect of the SGE for crossbred performance in the purebreds (p-value < 0.08). In other words, the log-likelihood test could not confirm the existence of a genetic correlation between SGE for ADG in the purebreds and crossbreds.

## Discussion

In this study, we show that SGE has a positive effect on phenotypic ADG in crossbreds. Moreover, we show that there is evidence of social genetic variation in purebreds, which is expressed as phenotypic ADG in crossbreds, and that this is favourably genetically correlated with the social genetic variation expressed as ADG in the purebreds. Thus, our results indicate that selection for SGE on ADG in purebreds in a nucleus farm environment with little competition for resources can improve ADG in crossbreds in a commercial environment.

### Effect of SGE on phenotypic ADG

We found an effect of SGE on phenotypic ADG of 0.37 with a 95% confidence interval of [− 0.04;0.78], conditioning on the average SD in starting weight across groups. This means that within a given group, the ADG of each pig will increase by 0.37 g/day, when the sum of its group mates’ SGE increases by 1 g/day. This is lower than the expectation, which was equal to 1 as the phenotype is a direct function of the ${\text{cSGE}}$ given by the equation ${\text{P}} = {\text{DGE}} + {\text{cSGE}} + {\text{e}}$ [2]. However, the average group size differed markedly between purebreds (10.8 and 11.9) and crossbreds (17.4), thus dilution effects may be present, which we were unable to account for. Although for the same purebreds as used in this study, previous studies could not identify dilution effects, based on a group size interval of 8–15 [4, 20]. In the presence of dilution effects, the sum of SGE of the group mates (${\text{cSGE}}$) along with its cumulative effect on the growth of group mates would be expected to decrease with increasing group size. Thus, the expectation for the regression coefficient might be in fact lower, in proportion to the relatively larger group size for crossbreds compared to purebreds. The results support our hypothesis that SGE estimated in purebred pigs have a positive effect on phenotypic ADG in crossbred pigs. This effect was found although the contrast between high and low social groups was relatively small at the phenotypic level—only 8 g/day (1015 g/day for pigs with high SGE and 1007 g/day for pigs with low SGE). This contrast was smaller than the 31 g/day expected based on the realized selection differential for SGE (re-estimated to 1.60 g/day/pig) and also smaller than the 16 ± 2.9 g/day expected based on re-estimated prediction error variances of sires and dams (following the approach by [7] in their Appendix 1). Retrospectively, the achieved contrast yielded an experimental power of 0.82 to detect the estimated regression coefficient as statistically significant from 0. Thus, this experiment had sufficient power to detect the observed contrast (as suggested by the p-value, see Table 2), whereas the power of previous studies was too small to account properly for environmental and/or crossbred effects [6, 7].

The relatively small realized phenotypic contrast in ADG may be explained partly by the realized selection intensity and partly by the genetic correlation between purebreds/selection environment and crossbreds/experimental environment. The realized selection intensity was lower than planned, since the actual number of selected sires was four times larger than planned (199 vs 50) due to availability on the artificial insemination (AI) station. Similarly, the actual number of selected dams was twice larger than planned (911 vs 400) due to management decisions at the nucleus farm. Given the realized selection intensity, the expected contrast was reduced from ~ 19 g/day to ~ 12 g/day. Furthermore, our results suggest that the genetic correlation between SGE in the purebreds and crossbreds and/or between the selection (nucleus) and experimental (production) environment was 0.52 and 0.34 in ${\text{L}}$ and ${\text{Y}}$, respectively. Thus, they were lower than 1, which reduces the expected phenotypic contrast in the crossbreds/experimental environment.

The regression coefficient of ADG on DGE (${\text{b}}_{{{\text{DGE}}}}$) was statistically significantly different from 0 and favourable (0.30). A favourable regression coefficient of DGE was expected since, previously, the genetic correlation for the DGE of ADG between purebreds and crossbreds was found to range from 0.53 to 0.99, e.g. [21–23].

### SGE and uniformity of pigs in groups

We found a negative regression coefficient of -0.26, statistically significantly different from 0, for the interaction between ${\text{cSGE}}$ and SD in starting weight on ADG. This indicates that a higher SD in starting weight reduces the effect of the ${\text{cSGE}}$ and thereby decreases the benefit of a positive SGE on ADG. In contrast, the effect of ${\text{cSGE}}$ on ADG will be greater in groups with pigs that are more uniform with regards to starting weight (Fig. 1). Thus, the results confirm our hypothesis that variation in starting weight is associated with social interactions among pigs within groups. No other study has previously reported such an interaction, but the influence of variation in body weight on growth has been investigated in several studies. The majority of these studies suggest that reducing the variation in body weight at the start of the finisher period does not affect the average growth performance until slaughter [24–27]. This is not in agreement with our study, as the main effect of variation in starting weight on ADG was -6.09 with a 95% confidence interval of [2.17;10.1] not including zero. This is consistent with the observation that variation in weight does not seem to have an effect on behaviour, since pigs are more aggressive towards each other when the variation in body weight within the group is smaller [11]. This may be because body weight is a determinant of competitive ability in pigs and the establishment and maintenance of dominance relationships are more likely to require assessment of competitive ability through fighting in weight-matched pigs [11]. If SGE on ADG reflect the ability to resolve conflicts with familiar pigs without aggression, as previously suggested [12, 13], then it is possible that SGE can have a larger effect on ADG under conditions where pigs are of similar weight and competitive ability.

### Genetic parameters in purebreds and crossbreds

We hypothesized that social genetic variance exists for ADG in crossbreds and that there is a favourable genetic correlation between SGE for ADG in purebred and crossbred pigs. Both are important because, if they are confirmed, then selection for SGE in purebreds is expected to result in improved performance in crossbreds. In this study, we estimated genetic parameters for ADG in purebreds and crossbreds, including social genetic covariances between purebreds and crossbreds.

The variances of SGE in the purebreds on crossbred performance ($\sigma_{S,L - YL}^{2}$ = 41.5 and $\sigma_{S,Y - YL}^{2}$ = 27.5) would indicate the presence of social genetic variance in the crossbreds, but they were not statistically significantly different from 0, and therefore cannot confirm that social interactions among crossbred pigs within groups are partly genetically determined. The magnitude of these social genetic variances was 5 to 7 times larger than the corresponding variances in the purebreds (5.9 in ${\text{L}}$ and 5.3 in ${\text{Y}}$). Thus, the relative magnitudes of social genetic variances for ADG in crossbred and purebred pigs are similar to the relative levels observed in previous studies [3, 5, 28, 29]. Similarly, the total genetic variances were, much larger in the crossbreds (5408 and 4508 in ${\text{L}}$-${\text{ YL}}$ and ${\text{Y}}$-${\text{ YL}}$, respectively) than those in the purebreds (3167 and 3972 in ${\text{L}}$ and ${\text{Y}}$, respectively), which reflects both the larger social genetic variances for performance in the crossbreds and the larger group size in the experimental environment (17.4) compared to the nucleus (10.8 and 11.9 for ${\text{L}}$ and ${\text{Y}}$, respectively).

Our results ($r_{g,L - YL}$ = 0.52 and $r_{g,Y - YL}$ = 0.34) also indicate (although not statistically significantly different from 0) that there is a favourable genetic correlation between SGE for ADG in purebreds and crossbreds. Therefore, the estimated genetic parameters in both ${\text{L}}$ and ${\text{Y}}$ support the result obtained in the regression analysis discussed above (Model 1), i.e. that selection for SGE estimated in purebreds will improve ADG in crossbreds. Estimated genetic correlations lower than 1 suggest either the presence of genotype-by-environment interactions for SGE [30] or the existence of non-additive genetic effects in combination with differences in allele frequencies between the two pure breeds, ${\text{L}}$ and ${\text{Y}}$. Genotype-by-environment interactions may occur due to either differences in the magnitude of genetic variation between the selection and experimental environments [31] or reranking of genotypes between environments [32].

The expected performance in the crossbreds based on purebred selection on DGE and SGE can be calculated based on the estimated genetic (co)variances (Tables 3 and 4). For selection on DGE only in the purebreds, the increase in crossbred performance is given by $\frac{{\sigma_{D,k;D,k - YL} }}{{\sigma_{D,k}^{2} }}$, where $k = \left\{ {L,Y} \right\}$, yielding an added 0.45 g/day and 0.53 g/day originating from ${\text{L}}$ and ${\text{Y}}$, respectively for each improvement in DGE in the purebreds of 1 unit. Due to the negative genetic correlation between DGE in the purebreds and SGE in the crossbreds, a negative response due to social interactions should be subtracted from this, namely $\frac{{\sigma_{D,k;S,k - YL} }}{{\sigma_{D,k}^{2} }}$, yielding -0.011 g/day and -0.02 g/day originating from ${\text{L}}$ and ${\text{Y}}$, respectively. In other words, if selection is only on DGE then there is only a small, yet negative, change in SGE in the crossbreds. Likewise, the expected performance in the crossbreds based on selection on SGE in the purebreds is given by $\frac{{\sigma_{S,k;D,k - YL} }}{{\sigma_{S,k}^{2} }}$ and $\frac{{\sigma_{S,k;S,k - YL} }}{{\sigma_{S,k}^{2} }}$. If selection is in the purebreds ${\text{L}}$, this yields an added ADG in the crossbreds of 13.9 g/day due to DGE and 1.4 g/day due to SGE, and if selection is in the purebreds ${\text{Y}}$, this yields an added ADG in the crossbreds of − 23.4 g/day due to DGE and 0.77 g/day due to SGE. In other words, the magnitudes of the estimated genetic (co)variances suggest that the selection for SGE in the purebreds will indeed be passed on to the crossbreds.

### Experimental design

Power to estimate genetic covariances precisely was lacking. The number of crossbred groups was 273, which may be sufficient to detect that the social genetic variance is statistically significantly different from 0 in an optimum design depending on its magnitude. An optimal design for the estimation of variances of SGE would comprise small groups of few families [19]. However, this type of design would not be of practical relevance for pig production. With large group sizes (15 or 30), Ødegård and Olesen [33] also found that social genetic variances were less accurately estimated with a random group composition than with a three-family structure. In this study, several half-sib families were represented within each group (on average 5.3 sires and 13.1 dams), approaching a random group composition, and group size was large (17.4 on average). Hence, 273 groups may be insufficient for accurate estimation of social genetic variance in a study such as this one. Thus, although the social genetic variances estimated in the crossbreds were relatively large (41.5 ± 28.2 and 27.5 ± 14.3), it is not surprising that they were not statistically significantly different from 0. On the contrary, it was possible to detect that social genetic variances in the purebreds were statistically significantly different from 0 (5.9 ± 2.0 and 5.3 ± 1.7), probably because of the much larger number of groups per breed (10,184 and 12,828 groups) combined with smaller group sizes (10.8 and 11.7).

This selection experiment was not designed with the purpose to estimate genetic variances of SGE, but rather to test for a significant difference in performance on offspring groups of high and low SGE. At the group level, pen effects and SGE are confounded, whereas the analyses at the individual level presented in this paper, allow for the separation of these. The best design to distinguish pen effects and SGE is a family design with two to three full sib families [19, 33], but such a design is not typical in pig production. Social genetic parameter estimates previously published have typically been based on groups of purebred pigs in a commercial setting e.g. ([3–5]), with groups consisting of several families, as in this experiment. In fact, in this experiment the average relatedness within groups in the crossbreds was 0.158, which was close to that in the purebreds (0.153 and 1.179 for Y and L respectively), which are typical commercial settings. Therefore, the group composition is not optimal for the estimation of genetic parameters, but it is comparable to similar studies. This experiment was conditional on the genetic level of SGE in purebreds and designed by maximising SGE genetic variance between purebred parents (the principle of the divergent selection within breeds). Thereby, the genetic (co-)variances between purebreds and crossbreds of SGE were designed to be larger than if selection had been obtained among random purebred parents. However, the genetic correlation $\left( {r_{S,k;S,YL} = \frac{{\sigma_{S,k;S,YL} }}{{\sqrt {\sigma_{S,k}^{2} \times \sigma_{S,YL}^{2} } }}} \right)$ should not be affected by this. In summary, we found that the actual design used in our study is appropriate for estimating purebred- crossbred genetic correlations for SGE, and it might be easily adopted to identify such purebred-crossbred correlations for other traits.

### Model comparison

The log-likelihood test could not confirm the existence of a genetic correlation between SGE for ADG in the purebreds and crossbreds. However, the expected performance based on the genetic parameter estimation was close to the expectation of 1 as opposed to the regression coefficient estimated with Model 1. We compared the linear regression coefficient (${\text{b}}_{{{\text{cSGE}}}}^{*}$) obtained based on Model 1 with regression coefficients calculated as a function of the genetic (co-)variances, i.e.: $\beta = \frac{{Cov\left( {X,Y} \right)}}{Var\left( X \right)}$. This corresponds to the expected correlated response in crossbreds to selection in purebreds. For ${\text{L}}$, this yields: $\beta_{L,L - YL} = \frac{{\sigma_{{{\text{S}},{\text{L}};{\text{S}},{\text{L}} - {\text{YL}}}} }}{{\sigma_{{{\text{S}},{\text{L}}}}^{2} }} = \frac{8.2}{{5.9}} = 1.39$ and similarly for ${\text{Y}}$, it yields: $\beta_{Y,Y - YL} = \frac{{\sigma_{{{\text{S}},{\text{Y}};{\text{S}},{\text{Y}} - {\text{YL}} }} }}{{\sigma_{{{\text{S}},{\text{Y}}}}^{2} }} = \frac{4.1}{{5.3}} = 0.77$. Thus, the aggregated mean of ${\text{L}}$ and ${\text{Y}}$ yields 1.08, which implies that SGE predicted in purebreds and crossbreds were nearly equivalent, whereas the corresponding regression coefficient estimated with Model 1 was 0.37 ± 0.21. Thus, the estimates of the genetic correlations and variances imply that the regression coefficient should be higher than that found with Model 1, where the upper confidence limit is equal to 0.37 + 1.96 × 0.21 = 0.79. This disagreement could be caused by the uncertainty in the results but also by the differences in average group size between purebreds and crossbreds. The average group size differed between purebreds (10.8 and 11.9) and crossbreds (17.4). Since no dilution effects were found in the purebreds based on a group size interval of 8–15 [4, 20], the sum of SGE of the group mates (${\text{cSGE}}$) along with its cumulative effect on the growth of group mates is expected to increase with increasing group size. Results from Model 1 show the opposite effect, i.e. the effect of ${\text{cSGE}}$ is lower than expectation (1) and results from Model 2 (1.08). This may imply the presence of dilution effects, which are not accounted for in Model 2 or in the selection of the purebreds, and it implies that SGE on growth are less important in larger group sizes. The disagreement between results from Model 1 and Model 2 may also be a consequence of crossbred information. Whereas Model 1 was based on DGE and SGE predicted in the two purebred parent populations, the DGE and SGE in Model 2 were predicted simultaneously in the purebred and crossbred populations. Thus, the difference between the estimated regression coefficient from Model 1 (${\text{b}}_{{{\text{cSGE}}}}^{*}$ = 0.37) and Model 2 (1.08) may indicate the need for crossbred information in purebred evaluation, which was also previously suggested in [34].

### Implications

The results from this study imply that selection for SGE is not only is possible, but it is also expected to result in a favourable response in ADG in crossbreds in a commercial environment even though selection is done in purebreds in a nucleus farm environment with little competition for resources. If higher SGE are indeed a reflection of less damaging behaviours among pigs within groups throughout the finisher period as suggested by some studies [12, 35], then selection for SGE is expected to lead to both higher growth and higher animal welfare in group-housed pigs. The negative interaction between the variation in starting weight and SGE implies that the benefits of selection for SGE will be more pronounced in production herds with a relatively high uniformity in starting weight within finisher pens. If selection for SGE on growth was implemented in pig breeding programs, a meaningful effect at the production level in commercial farms should be achievable after multiple generations of selection. If dilution is present for SGE on growth in crossbreds, then benefits on growth in commercial environments may be lower than expected as group sizes are commonly larger in commercial environments than in nucleus farms.

## Conclusions

In this study, we confirmed that SGE estimated using purebred information have a positive effect on phenotypic ADG in crossbreds, and that the largest effect is achieved when the within-group standard deviation of starting weight is small. For the average value of this standard deviation of starting weight within group, the individual ADG is expected to increase by 0.37 g/day when the sum of the group mates’ SGE increases by 1 g/day. Moreover, we have found indications of social genetic variation in purebreds (41.5 and 27.5 in ${\text{L}}$ and ${\text{Y}}$, respectively) for the expression of ADG in crossbreds, and this was favourably genetically correlated with the social genetic variation expressed as ADG in the purebreds (0.52 and 0.34 in ${\text{L}}$ and ${\text{Y}}$, respectively). Thus, the results indicate that selection for SGE on ADG in purebreds in a nucleus farm environment with little competition for resources can improve ADG in crossbreds in a commercial environment.

## Data Availability

The data analyzed during this study is not publicly available since it is owned by SEGES P/S.
